# Retraction: Matsunaka, T., et al. Temporal Variations of Polycyclic Aromatic Hydrocarbons in the Seawater at Tsukumo Bay, Noto Peninsula, Japan, during 2014–2018. *Int. J. Environ. Res. Public Health* 2020, *17*, 873

**DOI:** 10.3390/ijerph18041574

**Published:** 2021-02-07

**Authors:** IJERPH Editorial Office

**Affiliations:** MDPI, St. Alban-Anlage 66, 4052 Basel, Switzerland

The journal retracts the article “Temporal Variations of Polycyclic Aromatic Hydrocarbons in the Seawater at Tsukumo Bay, Noto Peninsula, Japan, during 2014–2018” cited above [1]. Following publication, the authors contacted the Editorial Office regarding significant errors in the calculation of PAH concentrations throughout the period of study. Corrected data are approximately 2.1–2.4 times higher than the PAH concentrations reported in the previous version.

Adhering to our standard operating procedures, an investigation was conducted that confirmed the extent to which changes were made in PAH concentrations. Because of the significant changes to the previously reported data, it was decided that a correction would not be appropriate. Therefore, the paper has been retracted from publication.

The authors agreed to this retraction.

The retraction was approved by the Editor-in-Chief of the journal.
